# A Deep Learning Radiomics Model based on Superb Microvascular Imaging for Non-Invasive Prediction of the Degree of Arteriolosclerosis in Patients With Chronic Kidney Disease

**DOI:** 10.2174/0115734056412035251027064309

**Published:** 2025-11-29

**Authors:** Yanhua Li, Xiaoling Liu, Chaoxue Zhang, Xiachuan Qin

**Affiliations:** 1 Department of Ultrasound, Beijing Anzhen Nanchong Hospital of Capital Medical University & Nanchong Central Hospital, Nanchong, Sichuan, China; 2 Department of Ultrasound, The First Afffliated Hospital of Anhui Medical University, Hefei, Anhui, China; 3 Department of Ultrasound, Chengdu Second People's Hospital, Chengdu, Sichuan, China

**Keywords:** Chronic kidney disease, Arteriolosclerosis, Ultrasound, Superb microvascular imaging, Deep learning radiomics, Heart disease

## Abstract

**Objective:**

This study aimed to develop and validate a deep learning radiomics (DLR) model based on superb microvascular imaging (SMI) for the non-invasive assessment of the severity of arteriolosclerosis in patients with chronic kidney disease (CKD).

**Materials and Methods:**

From June 2022 to December 2024, we prospectively recruited 326 CKD patients who underwent kidney biopsy across two medical centers. The enrolled patients were randomly allocated to the training or testing set in a 7:3 ratio. Deep learning (DL) features and radiomics features from SMI images were extracted, and after dimensionality reduction, they were used to establish deep learning radiomics (DLR) models. The performance of the proposed models was assessed through receiver operating characteristic (ROC) analysis and decision curve analysis (DCA).

**Results:**

Among the 326 CKD patients, 165 were positive for arteriolosclerosis and 161 were negative. In the training group, the area under the curve (AUC) values for the CDUS model,clinical model, radiomics model, DL model, and DLR model were 0.621 (0.547-0.695), 0.68 (0.611-0.749), 0.763 (0.703-0.823), 0.820 (0.767-0.874), and 0.840 (0.790-0.890), respectively. In the testing group, the AUCs were 0.677 (0.571-0.783), 0.776 (0.684-0.869), 0.727 (0.626-0.829), 0.779 (0.687-0.872), and 0.819 (0.735-0.903), respectively. The DLR model outperformed standalone radiomics, DL models, and the CDUS-based clinical model. The DCA validated the clinical utility of the DLR model.

**Conclusion:**

The DLR model utilizing SMI imaging can precisely and non-invasively assess the severity of arteriolosclerosis in CKD patients, which can assist physicians in formulating more favorable treatment plans for patients.

## 1 INTRODUCTION

Chronic kidney disease (CKD) is a highly prevalent chronic condition that serves as a substantial independent risk factor for adverse cardiovascular outcomes, including coronary heart disease, stroke, and heart failure [1]. According to survey results, there are currently 82 million adults with CKD in China [2]. By 2040, CKD is expected to become the fifth leading cause of death globally [3]. The incidence of CKD has reached the level of a pandemic and is continually increasing, becoming a major global medical, social, and economic burden. Given the high incidence and latency characteristics of CKD, real-time monitoring is crucial for treatment implementation and preventing its progression into end-stage renal disease (ESRD). Arteriolosclerosis is an important indicator of the severity of CKD [4]. Arteriolosclerosis progression may trigger or worsen kidney disease. Vascular rarefaction or dysfunction can lead to tissue ischaemia, compromise kidney haemodynamics, and drive fibrosis in the renal interstitium, ultimately contributing to CKD [5, 6]. It is also an important indicator affecting the prognosis of CKD [7-9]. During the pathological progression of CKD, renal arteriolosclerosis, characterised by intimal hyperplasia and thickening, narrows the luminal diameter. This elevated hemodynamic resistance in the renal microvasculature reduces vascular perfusion in the renal cortex [10, 11]. Pathologically, in early and mildly impaired CKD, the haemodynamic resistance of microvessels increases with changes in the renal cortical microvessels [12].

Colour Doppler ultrasound (CDUS) is the primary method to assess arteriolosclerosis in CKD [13]. Distinguishing blood flow signals from motion artifacts is difficult using CDUS [14]. The ability to detect low blood flow in the slow capillary vessels within the kidneys is relatively poor, even with more sensitive power Doppler ultrasonography; it can only detect blood vessels with a minimum diameter of 0.2 millimeters [15, 16]. In recent years, superb microvascular imaging (SMI) has been used as a new non-invasive blood flow imaging modality in clinical practice, effectively differentiating true microvascular flow signals from motion artifacts and noise, and displaying microvessels in the renal cortex with very high spatial resolution [17]. According to Gao's research, when displaying the cortical microvasculature of healthy kidneys, SMI technology demonstrates substantially superior sensitivity compared to conventional CDUS technology [18]. Therefore, SMI shows great potential for demonstrating the microcirculation of the kidneys.

Although ultrasound examination is widely used in the diagnosis of clinical diseases, the diagnostic performance of ultrasound largely depends on the clinical and professional knowledge of the diagnosing physician, and ultrasound diagnosis still faces key challenges, such as operator dependence, subjective image analysis, and the absence of reliable quantification methods [19]. Widespread attention has been drawn to artificial intelligence because of its remarkable capabilities in image recognition — diagnostic accuracy in medical imaging interpretation can be substantially enhanced, making diagnoses more objective [20-22]. Radiomics transforms medical images into explorable high-throughput quantitative features [23, 24], utilising information related to lesion phenotypes, such as shape, intensity, wavelets, and texture, to assess the heterogeneity of lesions. According to recent studies, biomarkers of subclinical renal alterations can be accurately identified using ultrasound radiomics analysis [25, 26]. Deep learning radiomics (DLR) is an innovative approach that enables end-to-end learning and automatically discovers multiple levels of representations for specific predictive tasks. The DLR process involves inputting raw medical images, learning feature representations, quantifying information within the images, and using multilayer feature discovery for predictive task vectors [27]. Therefore, we attempted to establish a DLR model based on SMI for non-invasive assessment of arteriolosclerosis in CKD.

## 2 MATERIALS AND METHODS

### 2.1 Patient Selection

The research was conducted following the ethical principles outlined in the Declaration of Helsinki and and was granted ethical approval by the hospital committee of the First Affiliated Hospital of Anhui Medical University (PJ2022-11-29).. As this research was retrospective, the requirement for informed consent was waived, and patient confidentiality was strictly maintained. From June 2022 to December 2024, we consecutively recruited 326 CKD patients from two medical institutions who underwent renal puncture biopsy as study subjects. Among them, there were 145 cases of IgAN (IgA nephropathy), 90 cases of membranous nephropathy, 20 cases of mesangial proliferative glomerulonephritis, 11 cases of minimal change nephropathy, 13 cases of tubulointerstitial nephritis, 11 cases of hypertensive nephropathy, 12 cases of diabetes nephropathy, 6 cases of lupus nephritis, 9 cases of glomerular podocytosis, and 9 cases of other types of nephropathy. The inclusion criteria were as follows: (1) patients containing more than 10 glomeruli and 2 arteries under optical microscopy, (2) age over 18 years, and (3) a distance of less than 5 cm between the kidney and the skin. The exclusion criteria were: (1) acute kidney injury or heart valve disease, (2) urinary tract obstruction, (3) the presence of cysts or tumors in the kidney, and (4) Doppler patterns suggesting renal artery stenosis [28]. The research flowchart is shown in Fig. (**1**).

Age and glomerular filtration rate (eGFR) are well-known key factors closely associated with arteriosclerosis [29], and we included age and eGFR in establishing a clinical model.

### 2.2 Pathological Grading

An experienced nephrologist and an interventional ultrasound physician cooperated to perform a kidney biopsy of the target kidney under ultrasound guidance. For each biopsy specimen, routine examinations were conducted using optical microscopy, immunofluorescence, and electron microscopy. Tissue sections were stained with hematoxylin-eosin staining, periodic acid-Schiff reaction, Masson's trichrome, and periodic-acid silver methenamine staining, and the biopsy specimens included the presence of at least 10 glomeruli and 2 arteries. Two experienced renal pathologists (with 10 and 12 years of experience) reviewed the kidney biopsy specimens under an optical microscope at 100-400x magnification for diagnosis, and they graded arteriolosclerosis, defining Intimal thickening<thickness of media as arteriolosclerosis negative and Intimal thickening≥thickness of media as arteriolosclerosis positive [4].

### 2.3 Ultrasound Image Collection

SMI images were collected by three experienced sonographers (with abdominal ultrasound experience of 6, 10, and 16 years, respectively), using a Canon Aplio 700 diagnostic system (Canon Medical Systems, Otawara, Japan) equipped with a 3.5 MHz convex array probe (i8C1). Patients were instructed to fast for more than 8 hours prior to the ultrasound examination. The ultrasound probe was gently positioned in the flank in the form of an oblique projection, and a 3.5 MHz probe was used for ultrasound examination of the target kidney. The probe was placed on the posterior axillary line, and its position and angle were adjusted to obtain a maximum longitudinal ultrasound image of the target kidney. After the maximum longitudinal section of the kidney was obtained, the settings of the ultrasound diagnostic instrument were switched to color SMI mode, and the specific area of interest box of SMI was placed on the whole kidney to obtain the optimal blood flow section of the kidney. The settings included a mechanical index of 1.6, a frame rate ranging between 5 and 25 frames per second, and a dynamic range of 65-75 dB. The SMI speed was set at 3.5 cm/s. To optimize imaging of renal blood flow, patients were instructed to briefly hold their breath during the scan.

### 2.4 Construction of CDUS Model

Pre-puncture CDUS data of the kidney were collected from patients as follows: kidney length, width, cortical thickness, renal echo, peak systolic velocity (PSV) of the main renal artery (MRA), resistance index (RI) of the main renal artery (MRA), and PSV and RI of the segmental renal artery (SRA) (**Supplementary Document 1**).

Univariable logistic regression analysis was performed to analyze the association between CDUS parameters and the presence of arteriolosclerosis, and the relevant factors obtained from the aforementioned univariable regression analysis (*p*<0.1) were subjected to multivariable logistic regression analysis to identify the significant independent predictive factors associated with arteriolosclerosis and further establish a clinical diagnostic model.

### 2.5 Radiomics Feature Extraction

All ultrasound images were saved in DICOM format after being directly exported from the ultrasound diagnostic system. First, we imported the SMI images of the target kidney into the ITK-SNAP medical image segmentation software (version 3.8.0 http://www.itksnap.org/pmwiki/pmwiki.php?n=Downloads.SNAP3), labeling the target kidney cortex as a region of interest (ROI). Then, a specialized open-source package (PyRadiomics, v3.1.0) was used to extract radiomic features from the SMI images, extracting 2D shape, first-order features, gray level co-occurrence matrix (GLCM), gray level size zone matrix features (GLSZM), gray level run-length matrix features (GLRLM), neighborhood gray-tone difference matrix features (NGTDM), gray level dependence matrix (GLDM), wavelet transform, squared, and gradient features (https://pyradiomics.readthedocs.io/en/latest/features.html). A random selection of 50 patient images was re-segmented by the same radiologist and an additional radiologist (12 years of ultrasound experience) to determine intra- and inter-observer reliability using intraclass and intergroup correlation coefficients (ICC). Only features with an ICC≥0.75 were retained for subsequent analysis.

### 2.6 Deep Learning Feature Extraction

Two experienced sonographers (with 10 and 16 years of kidney ultrasound examination experience, respectively) used open-source software Labelme (https://github.com/wkentaro/labelme) to mark the ROI with rectangular bounding boxes. Data were normalized and standardized to adjust image values to a range of 0-1 before being input into the network model, and the image size was adjusted to 244×244. We used a pre-trained model for feature extraction, leveraging SimSiam self-supervised learning [30]. The ResNet18 model, trained on 120,000 ultrasound images as a pre-trained model, significantly reduces the time and resources required to train the model from scratch while also improving its generalization ability. The pre-trained model provides a good starting point for initializing model weights, accelerating convergence, and enabling the model to demonstrate high performance during the early stages of training, thereby enhancing the generalization capability of the network model (**Supplementary Document 1**). During training, the complete ResNet18 was used for binary classification training, and after training, the fully connected layer and softmax layer of ResNet18 were removed, using the output values of the final layer nodes as deep learning (DL) features to extract deep features from the input ultrasound images and generate 2048 DL features.

### 2.7 Establishment of Machine Learning Model

We standardized the data using Z-score normalization after integrating radiomics features and DL features, performed significance analysis on the DLR features using the Mann-Whitney U test with a *p*-value threshold of<0.05, conducted Pearson correlation analysis, defined redundant features as those exceeding a threshold of 0.6, and eliminated them, and finally performed least absolute shrinkage and selection operator (LASSO) analysis. We used the CatBoost algorithm to model and analyze the radiomics features obtained from LASSO. Model parameter optimization was conducted using grid search and five-fold cross-validation methods. The best-performing ML model's feature importance was visualized using SHapley Additive exPlanation (SHAP). SHAP values derive from cooperative game theory, offering a mathematical method for fairly distributing expenditures among players. SHAP assigns values to features based on their relationship with model outputs, quantifying the importance of each feature. This process enhances transparency and interpretability in machine learning (ML) models by attributing specific contribution scores to individual features [31]. Figure. (**2**) shows the workflow of the SMI-based DLR model.

### 2.8 Statistics

Statistical analysis was conducted using SPSS version 25.0 (IBM Corp., Armonk, NY, USA) and Python 2.7 (Python Software Foundation, Beaverton, OR, USA). Continuous variables were assessed for normality and homogeneity of variance. Depending on the distribution, either an independent samples t-test (for normally distributed data) or the Mann-Whitney U test (for non-normally distributed data) was applied. Categorical variables were compared using chi-square tests. *P*< 0.05 was deemed statistically significant.

## 3 RESULTS

### 3.1 Clinical Characteristics of Patients

A total of 326 patients with CKD who underwent kidney biopsy were recruited for this study, including 175 males and 151 females, with an average age of 43.0±13.3 years. Among them, 165 CKD patients were arteriolosclerosis positive, and 161 CKD patients were arteriolosclerosis negative. The training group consisted of 228 patients (119 males and 109 females), with an average age of 43.5±13.4 years, of whom 115 CKD patients were arteriolosclerosis positive and 113 were arteriolosclerosis negative. The test group included 98 patients (56 males and 43 females), with an average age of 42.1±13.2 years, of whom 50 CKD patients were arteriolosclerosis positive and 48 were arteriolosclerosis negative. The clinical baseline characteristics of CKD patients are shown in Table **1**.

### 3.2 Diagnostic Performance of Clinical Model

The clinical model had an area under the curve (AUC) of 0.68 (0.611-0.749) in the training group, with an accuracy of 0.64, a sensitivity of 0.835, a specificity of 0.442, a positive predictive value (PPV) of 0.604, and a negative predictive value (NPV) of 0.725. In the test group, the AUC was 0.776 (0.684-0.869), with an accuracy of 0.714, a sensitivity of 0.8, a specificity of 0.625, a PPV of 0.69, and an NPV of 0.75 (Table **2**).

### 3.3 Diagnostic Performance of the CDUS Model

Univariable analysis and multivariable logistic regression analysis showed that the PSV of MRA was an independent predictive factor for arteriolosclerosis in the CDUS model. Furthermore, we established a clinical model to predict CKD arteriolosclerosis based on the PSV of the MRA, with an AUC of 0.621 (0.547-0.695) in the training group, an accuracy of 0.623, a sensitivity of 0.628, a specificity of 0.617, a PPV of 0.617, and an NPV of 0.628. In the test group, the model achieved an AUC of 0.677 (95% CI: 0.571-0.783). The accuracy was 0.663, with a sensitivity of 0.521 and a specificity of 0.800. The PPV was 0.714, while the NPV reached 0.635 (Table **2**).

### 3.4 Diagnostic Performance of the Radiomics Model

A total of 1746 SMI radiomics features were extracted from SMI images. Inter-observer and intra-observer analyses, and univariable correlation analysis indicated significant differences in 1325 SMI radiomics features between the two groups. The radiomics feature data were standardized using Z-score; a univariable test was performed for significance analysis, with a threshold of *p*<0.05, resulting in 61 remaining features. Pearson correlation analysis was performed to identify feature pairs with a correlation coefficient greater than 0.6. One feature was removed from each pair of highly correlated features, leaving 3 features: wavelet-LHL_glcm_Correlation, wavelet-HLL_glrlm_HighGrayLevelRunEmphasis, and wave let-HLL_glszm_GrayLevelNonUniformityNormalized.

We constructed a radiomics model using the CatBoost algorithm, achieving an AUC of 0.763 (0.703-0.823) in the training group, with an accuracy of 0.697, a sensitivity of 0.713, a specificity of 0.681, a PPV of 0.695, and an NPV of 0.700. In the test group, the AUC was 0.727 (0.626-0.829), with an accuracy of 0.704, a sensitivity of 0.580, a specificity of 0.833, a PPV of 0.784, and an NPV of 0.656 (Table **2**).

### 3.5 Diagnostic Performance of DL Model

The SMI images were processed to extract 2048 features using DL methods. A univariable test was conducted for significance analysis with a threshold of *p*<0.05, resulting in 280 remaining features. Pearson correlation analysis was then carried out to find feature pairs with a correlation coefficient greater than 0.6. One feature was removed from each pair of highly correlated features, resulting in 32 remaining features. Finally, the LASSO with alpha=0.5 was applied, resulting in 21 remaining features: DL3, DL23, DL24, DL32, DL41, DL277, DL325, DL367, DL467, DL546, DL584, DL608, DL639, DL642, DL830, DL864, DL1126, DL1383, DL1562, DL1597, and DL1922.

We developed a DL model based on the CatBoost algorithm. In the training cohort, the model demonstrated an AUC of 0.820 (95% CI: 0.767-0.874), along with an accuracy of 0.754. The sensitivity and specificity were 0.800 and 0.708, respectively, while the PPV and NPV were 0.736 and 0.777. When evaluated on the test group, the model achieved an AUC of 0.779 (95% CI: 0.687–0.872) and an accuracy of 0.735. Notably, the sensitivity dropped to 0.540, but the specificity increased to 0.938. The PPV and NPV in the test set were 0.900 and 0.662, respectively (Table **2**).

### 3.6 Diagnostic Performance of DLR Model

**Fig. (3):**
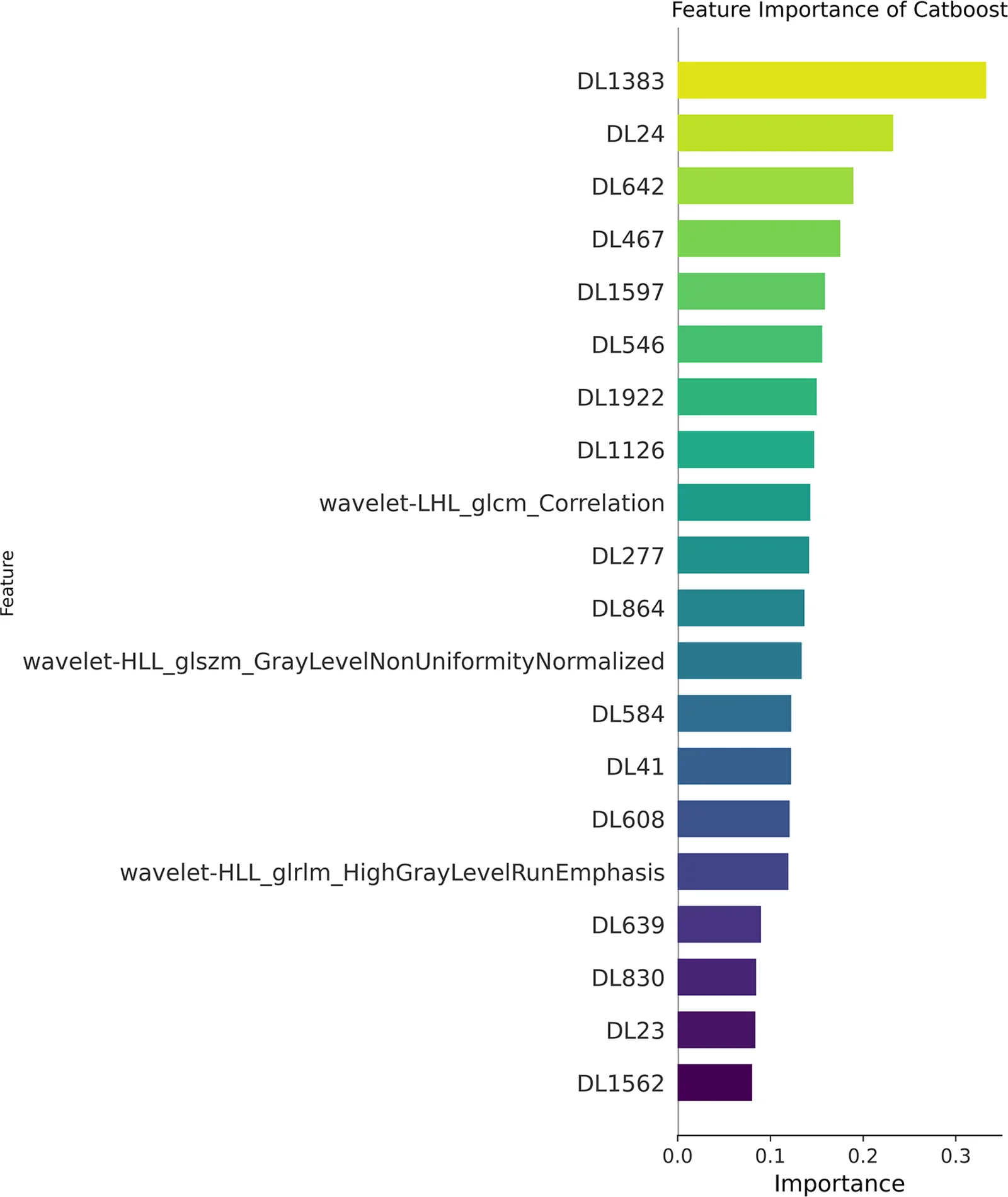
The key characteristics of the DLR model. **Note: ** DLR: Deep Learning Radiomics.

**Fig. (4):**
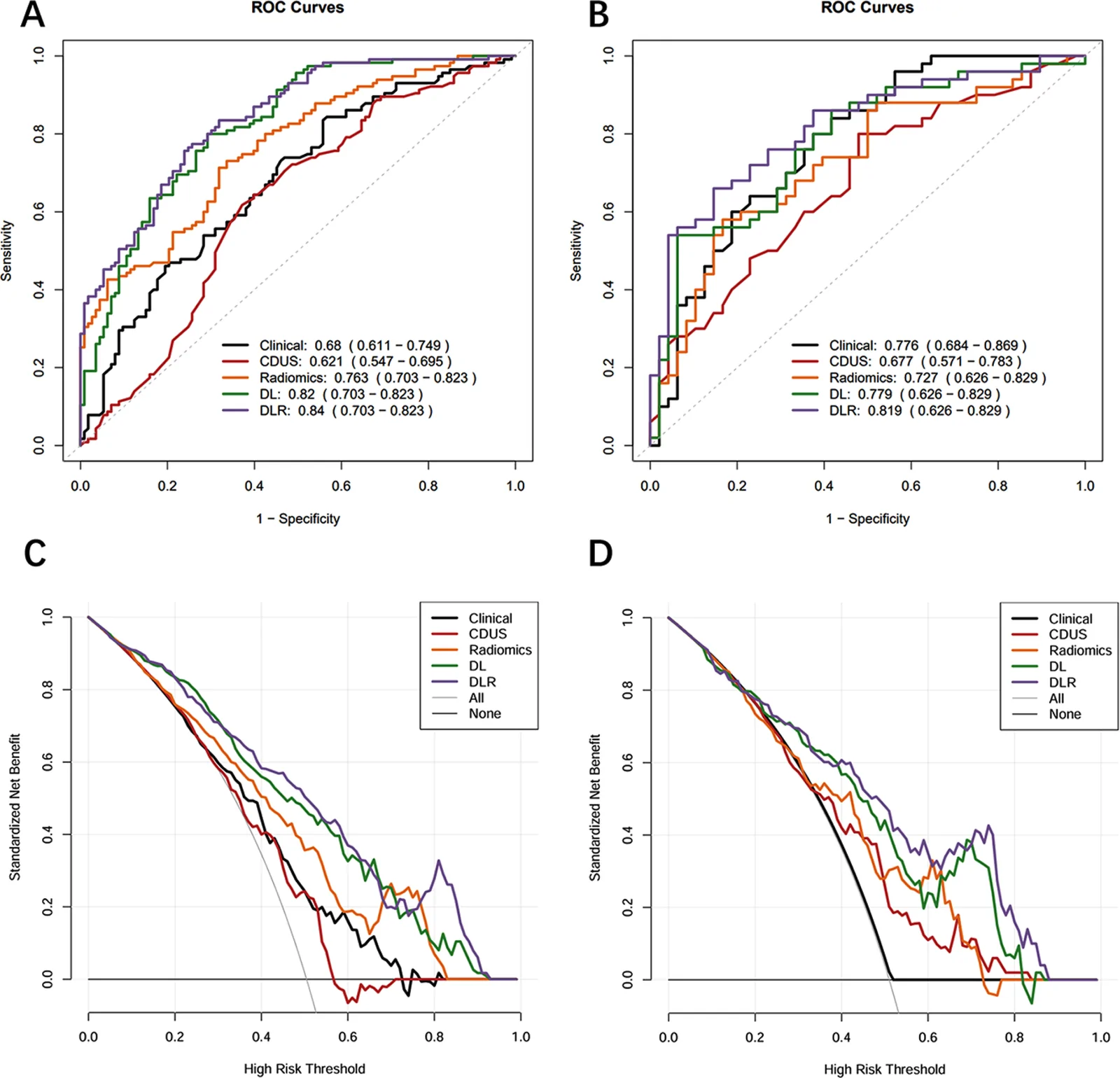
Performance evaluation of the four models: (**A**, **B**) ROC and DCA in the training group. (**C**, **D**) ROC and DCA in the testing group. **Note: ** DL = Deep Learning; DLR = Deep Learning Radiomics; ROC = Receiver Operating Characteristic; DCA = Decision Curve Analysis.

**Fig. (6):**
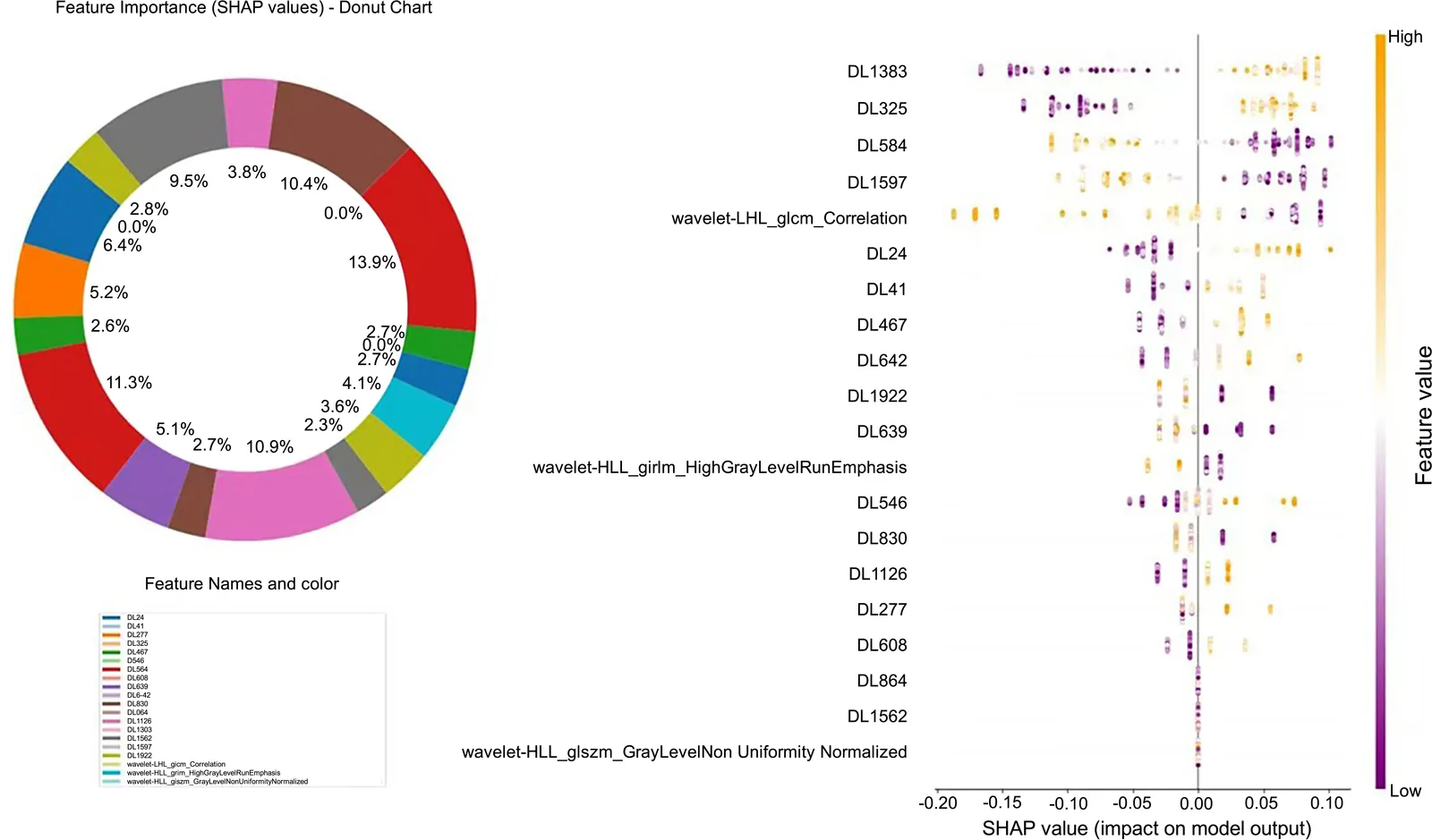
SHAP analysis visualization:the SHAP Beeswarm diagram and the SHAP donut chart. **Note: ** SHAP: SHapley Additive exPlanation.

By combining the 3 radiomics features and 21 DL features that showed significance, we performed dimensionality reduction using LASSO, ultimately identifying 21 key features (Fig. **3**). We then constructed a DLR model using the CatBoost algorithm, achieving an AUC of 0.840 (0.790-0.890) in the training group, with an accuracy of 0.759, a sensitivity of 0.757, a specificity of 0.761, a PPV of 0.763, and an NPV of 0.754. In the test group, the AUC was 0.819 (0.735-0.903), with an accuracy of 0.755, a sensitivity of 0.660, a specificity of 0.854, a PPV of 0.825, and an NPV of 0.707 (Table **2** and Fig. **4**). Meanwhile, DCA curves in both the training and test groups demonstrated that CatBoost had a higher overall net benefit compared to the other three models (Fig. **4**). Figure (**5**) shows the confusion matrices of the diagnostic performance in the DLR model in the training and testing groups. SHAP visualizations enhanced the interpretability of the DLR model, making the model more intuitive and interpretable. From the SHAP results, we found that among the top 5 features that significantly impacted the results, there were 4 DL features and 1 radiomics feature (Fig. **6**). The SHAP summary bar graph displayed the average SHAP values, evaluating feature contributions to the model, while the SHAP donut chart visually demonstrated the impact intensity of each feature on model predictions.

## 4 DISCUSSION

Renal perfusion is abundant, and renal blood flow typically accounts for 25% of the cardiac output [32].In the progression of CKD, arteriolosclerosis leads to gradual proliferation and thickening of the renal microvascular intima and other microvascular changes; a reduction in lumen diameter increases microvascular resistance; and increased intrarenal vascular resistance results in decreased microvascular perfusion of the renal cortex [11]. Changes in microvascular structure may also affect organ blood flow reserve [33]. Renal arteriolosclerosis is an independent predictor of CKD [4]. Therefore, developing a simple, non-invasive diagnostic model to evaluate the microvascular status of patients with CKD has become an urgent priority. In this retrospective study, we extracted DLR features from the SMI images of patients to establish an ML model for non-invasively evaluating the microvasculature in CKD, which demonstrated favourable discriminatory power in both the training (AUC=0.840) and test groups (AUC=0.819). To our knowledge, this is the first study using a DLR model based on SMI to predict the presence of arteriolosclerosis in CKD.

Arteriolosclerosis leads to thickening of the intima, narrowing of the lumen in small arteries and arterioles [34], and damage to the circulatory reserve of organs, resulting in reduced blood flow [32]. SMI represents a breakthrough in ultrasound Doppler imaging by leveraging a cutting-edge clutter suppression technique to isolate blood low signals from artifacts caused by tissue motion [18, 35]. Compared with conventional CDUS, SMI delivers higher frame rates, reduced clutter, and minimised motion artifacts. Additionally, it demonstrates greater sensitivity in detecting low-velocity blood flow and provides more detailed microvascular information. Notably, the results showed strong consistency with those of CEUS [34]. Therefore, we chose SMI imaging to evaluate the degree of arteriolosclerosis in CKD. However, the characteristics that can be detected in the kidneys by the human eye are limited; many internal details of the images are not recognisable to the naked eye. Unlike conventional CDUS, SMI enables the extraction of microfeatures from ROIs in medical imaging, converting lesion-related pathophysiological information into mineable high-dimensional data [36]. Our radiomics model achieved an AUC of 0.763 for the training group and 0.727 for the testing group, where the handcrafted features comprised only basic features, such as shape, greyness level, or texture information, making it difficult for the radiomics model based on radiomics features to meet clinical needs [37]. This study employs a ResNet18-based DL approach for feature extraction, leveraging DL's ability to uncover intricate patterns through its hidden layers without relying on predefined features. By integrating DL, radiomics can capture task-specific complex structures with enhanced depth and sophistication [38]. The research yielded a selection of 3 radiomics and 18 deep learning features, all of which were found to be complementary rather than overlapping in their information. Furthermore, we integrated the radiomics and DL features, obtaining results with a training group AUC of 0.840 and a testing group AUC of 0.819 for the DLR model, demonstrating a better performance than either the radiomics or DL methods alone. Being superior to traditional clinical models and CDUS models, the DR model has strong processing capabilities for high-throughput data, and can identify biological markers of subclinical changes based on SMI images, thereby comprehensively quantifying the arteriolosclerosis of renal microvasculature.

The SHAP interpretability method exhibits superior computational performance, higher local accuracy, and higher consistency, making it preferable to other interpretation methods [31]. SHAP offers a unified approach for interpreting DL models by quantifying feature importance through an increase in the prediction error when features are perturbed. By leveraging the SHAP, we can clarify the intricate network architecture of DL methods, which often obscures the interpretability of model outcomes. This framework enables researchers to probe the internal mechanisms of artificial intelligence models and evaluate their biological plausibility. Ultimately, these insights can help boost the confidence of both physicians and patients in the model's predictions [39]. Our DLR model identified 21 key features. From the SHAP results, we found that among the top 5 features that significantly influenced the outcome, there were 4 DL features and 1 radiomic feature. The results indicated that both DL and radiomic information contributed to the model, further validating the advancement of our DLR model.

### 4.1 Study Limitations

This study had several limitations. Firstly, because the patients with CKD stages 3B-5 were highly unlikely to undergo renal biopsy, most patients had CKD stage 1 or 2 in our study. For future research, we plan to collect and expand on the number of patients with CKD stages 3B-5. Secondly, the relatively small sample size might have led to biased results. Additionally, because all data were obtained from devices manufactured by a single vendor, the findings may not be broadly applicable to images acquired using devices from other manufacturers.

## CONCLUSION

In summary, we proposed a DLR model based on SMI for the non-invasive prediction of arteriolosclerosis in patients with CKD. The results showed that the DLR model based on SMI can effectively and non-invasively predict the presence of arteriolosclerosis in CKD and has good clinical applicability, providing an easy-to-use, personalised tool for the non-invasive diagnosis of arteriolosclerosis in CKD.

## Figures and Tables

**Fig. (1):**
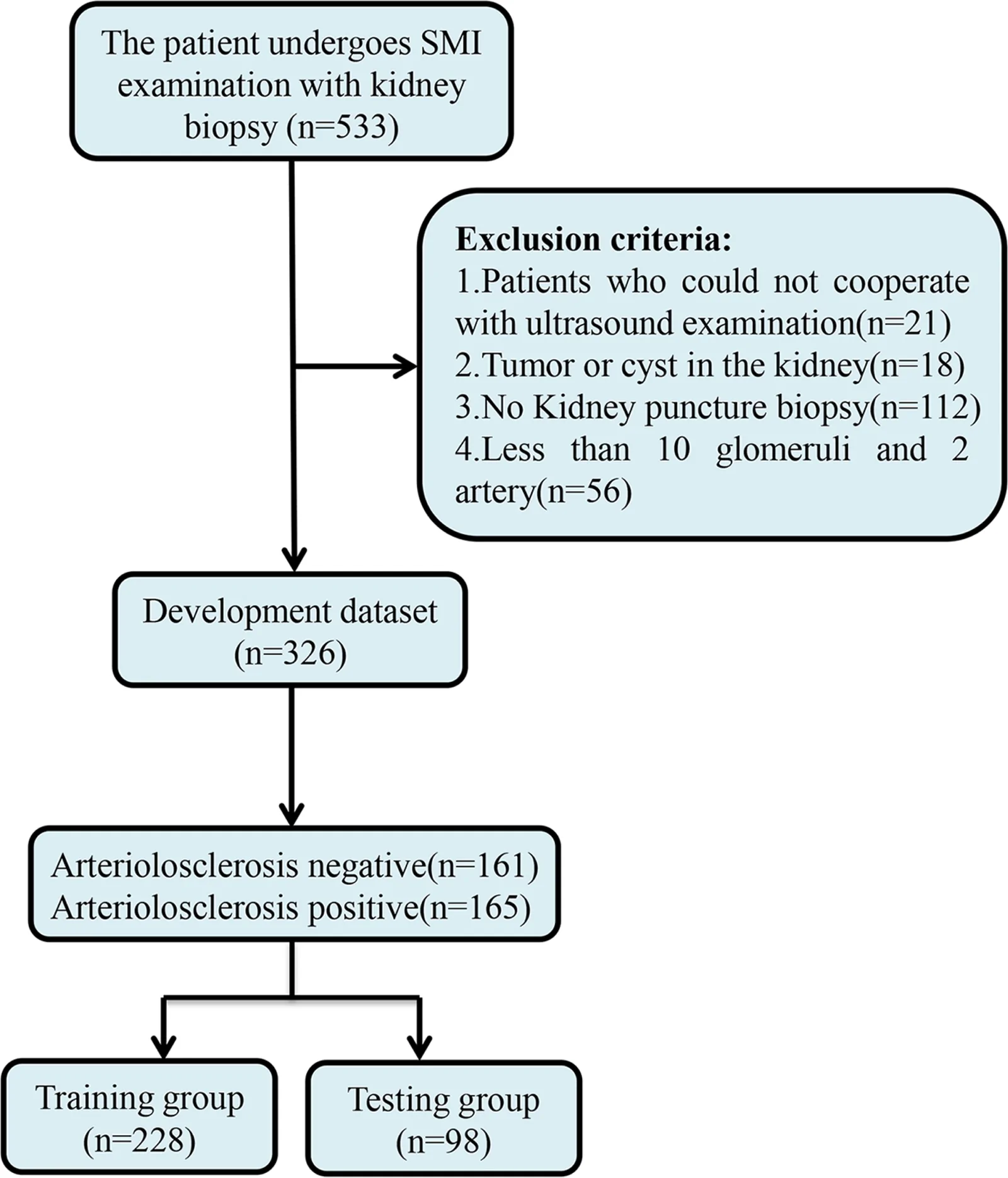
Patient enrollment and selection process. **Abbrevitaion:** SMI: Superb Microvascular Imaging.

**Fig. (2):**
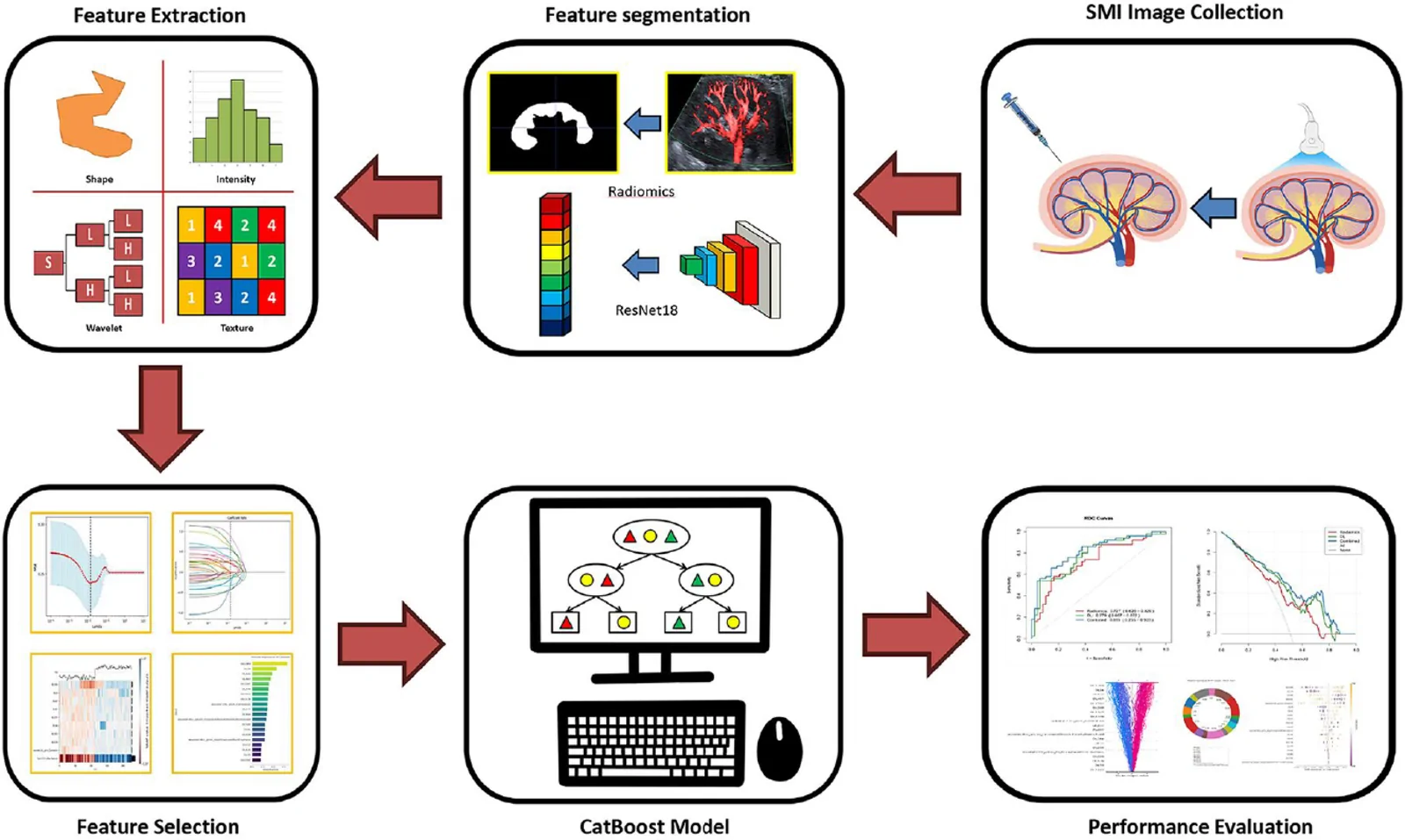
The workflow of the DLR analysis. **Note: ** DLR: Deep Learning Radiomics.

**Fig. (5):**
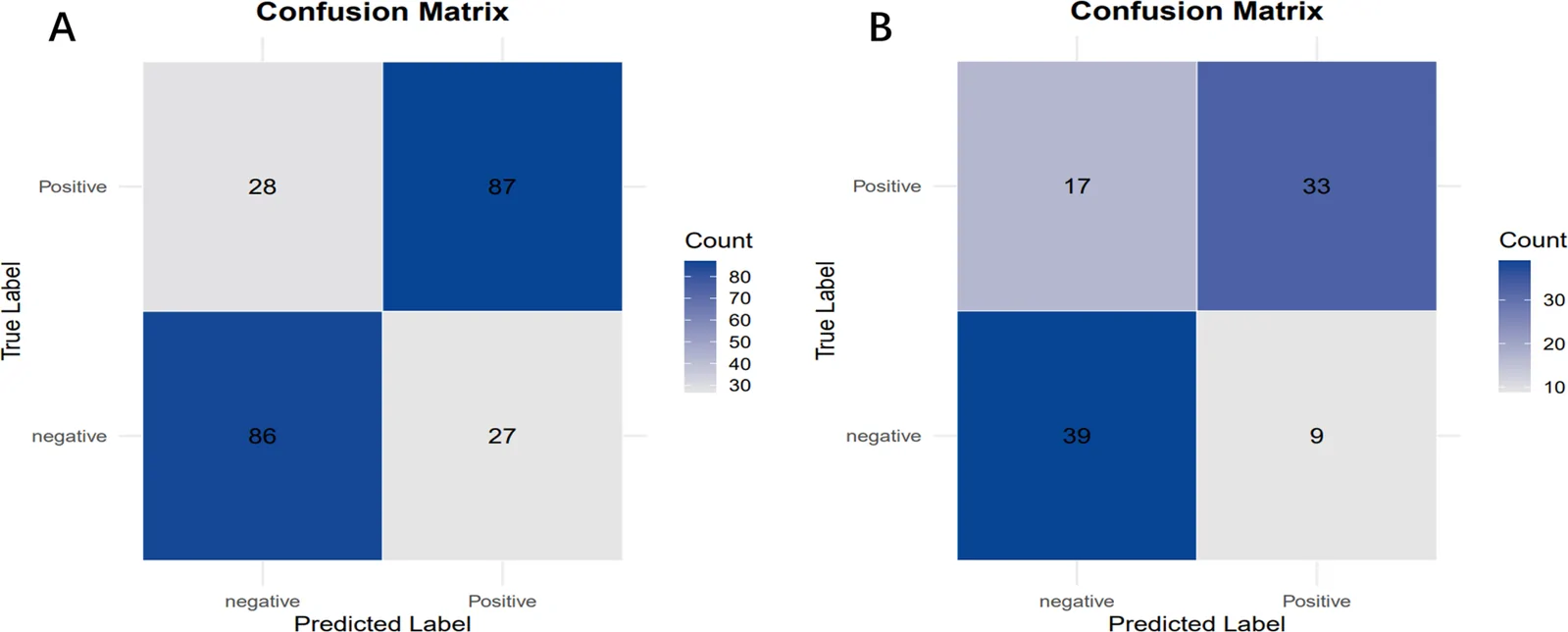
Confusion matrices of the diagnostic performance in the DLR model in the training and testing groups. **Note: ** DLR: Deep Learning Radiomics.

**Table 1:** Clinical factors in the training and testing groups.

Clinical Features	Training Group (n=228)	Testing Group (n=98)
Age (years)	43.5±13.4	42.1±13.2
Gender (Male**/**Female)	119/109	56/43
Kidney length (mm)	106.2±8.9	105.9±8.9
Urea (mmol/L)	6.5±2.8	6.3±3.3
Uric acid (μ mol/L)	378.9±97.7	384.0±112.4
24-hour proteinuria (g/24h)	2.9±3.7	2.5±3.2
Creatinine (μ mol/L)	98.1±64.2	94.5±69.1
eGFR(ml/min.1.73m^2^)	88±32.4	94.3±31
IF degree (≤ 25%**/**<25%)	163/65	71/27
Arteriosclerosis(positive**/***negative*)	115/113	50/48

**Note: **Calculate eGFR using CKD-EPI equation, IF= interstitial fibrosis.

**Table 2:** The diagnostic performance of the CDUS model, clinical model, radiomics model, DL model and DLR model in training and testing group.

-	AUC	ACC	SEN	SPE	PPV	NPV
**Training Group** CDUS model	0.621(0.547-0.695)	0.623	0.628	0.617	0.617	0.628
Clinical model	0.68(0.611-0.749)	0.64	0.835	0.442	0.604	0.725
Radiomics model	0.763(0.703-0.823)	0.697	0.713	0.681	0.695	0.700
DL model	0.820(0.767-0.874)	0.754	0.800	0.708	0.736	0.777
DLR Model	0.840(0.790-0.890)	0.759	0.757	0.761	0.763	0.754
**Testing Group** CDUS model	0.677(0.571-0.783)	0.663	0.521	0.800	0.714	0.635
Clinical model	0.776(0.684-0.869)	0.714	0.8	0.625	0.69	0.75
Radiomics model	0.727(0.626-0.829)	0.704	0.580	0.833	0.784	0.656
DL model	0.779(0.687-0.872)	0.735	0.540	0.938	0.900	0.662
DLR Model	0.819(0.735-0.903)	0.755	0.660	0.854	0.825	0.707

**Abbreviations: **ACC, accuracy; AUC, area under the receiver operating characteristic curve; NPV, negative predictive value; PPV, positive predictive value; SEN, sensitivity; SPE, specificity.

## Data Availability

All the data and supporting information are provided within the article.

## LIST OF ABBREVIATIONS

DLR: Deep learning radiomics
SMI: Superb microvascular imaging
CKD: Chronic kidney disease
DL: Deep learning
DCA: Decision curve analysis
ESRD: End-stage renal disease
eGFR: Age and glomerular filtration rate
PSV: Peak systolic velocity
MRA: Main renal artery
SRA: Segmental renal artery

## STANDARDS OF REPORTING

STROBE guidelines were followed.
