# Supplementary material for: A Deep Learning Radiomics Model based on Superb Microvascular Imaging for Non-Invasive Prediction of the Degree of Arteriolosclerosis in Patients With Chronic Kidney Disease
Source: Curr Med Imaging. 2025 Nov 29;22:E15734056412035. doi: 10.2174/0115734056412035251027064309 (PMC13579553; doi:10.2174/0115734056412035251027064309)
Supplement: Supplementary file 1 [file CMIM-22-E15734056412035_SD1.pdf]

1875-6603/26 *Send Orders for Reprints to reprints@benthamscience.net*

BENTHAM SCIENCE

CrossMark

Current Medical Imaging

Content list available at: https://benthamscience.com/journals/cmir

Supplementary Material

A Deep Learning Radiomics Model Based on Superb Microvascular Imaging for Non-Invasive Prediction of the Degree of Arteriolosclerosis in Patients With Chronic Kidney Disease

Yanhua Li[1,#], Xiaoling Liu[1,#], Chaoxue Zhang[2,*] and Xiachuan Qin[3,*]

[1]*Department of Ultrasound, Beijing Anzhen Nanchong Hospital of Capital Medical University & Nanchong Central Hospital, Nanchong, Sichuan, China*

[2]*Department of Ultrasound, The First Afffliated Hospital of Anhui Medical University, Hefei, Anhui, China*

[3]*Department of Ultrasound, Chengdu Second People's Hospital, Chengdu, Sichuan, China*

The included color Doppler ultrasound indicators include

1. Kidney length: the length of the target kidney measured in the maximum longitudinal section; 2. Kidney width: the width of the target kidney measured at the maximum longitudinal section; 3. Renal cortical thickness: the cortical thickness of the target kidney measured in the maximum longitudinal section; 4. Renal cortical echo: the renal cortical echo was compared with the liver / spleen echo. The normal renal cortical echo was defined as the renal echo was lower than the liver / spleen echo. The enhanced renal cortical echo was defined as the echo of the renal cortex was equal to or stronger than the echo of the liver / spleen parenchyma. The renal cortical echo intensity was divided into the enhanced renal cortical echo and the normal renal cortical echo; 5. Peak systolic velocity (PSV) of the main renal artery (MRA): images were obtained in color Doppler and pulse Doppler modes. The ultrasound probe was gently positioned in the flank in the form of oblique projection, and the kidney was visualized as a longitudinal image. The Doppler sampling volume was placed on the trunk of the renal artery of the target kidney, and the PSV of MRA was measured; 6. RI of MRA: the RI of MRA is calculated according to the peak systolic velocity (PSV) and end diastolic velocity (EDV) of the main renal artery measured for an average of 2-3 times, ri= (psv-edv) /psv; 7. The PSV of segmental renal artery (SRA) was obtained from two to three representative proximal PSVs of the target kidney (the first vascular branch of MRA), and the PSV of SRA was obtained; 8. RI of SRA: the RI of SRA is obtained by measuring PSV and EDV of SRA for an average of 2-3 times.

Training Strategy for Deep Learning Models

Efficient batch processing of training and validation datasets was achieved using the PyTorch framework's data loader. The use of data loaders not only improves the efficiency of data loading, but also significantly reduces the time required for CPU to GPU data transfer through its multi-threaded processing mechanism. In addition, in order to further enhance the generalization ability of the model, data augmentation techniques such as random cutting, horizontal rotation, vertical rotation, center rotation, changing size, resampling, and increasing noise have been introduced. These techniques help models learn richer and more robust feature representations by increasing the diversity of data.

This experiment adopted a distributed training method, which enables the model to be trained in parallel on multiple GPUs, greatly improving the efficiency of training. By reasonably configuring the parameters - dist url and - dist backed, an efficient distributed training environment was built, achieving balanced allocation and execution of training tasks across multiple computing nodes. In terms of optimization algorithms, stochastic gradient descent (SGD) was chosen as the main optimization tool. The advantage of SGD lies in its simplicity, efficiency, and ease of implementation and adjustment. Due to uneven training samples, we use Focal Loss to balance difficult samples. The original intention of designing this loss function is to reduce the impact of easy to classify samples on the loss function, while increasing the model's attention to difficult to classify samples. This strategy effectively solves the model bias problem caused by class imbalance [1]

Adjusting the learning rate is also a key step in this experiment. This experiment adopted a strategy of dynamically adjusting the learning rate, with an initial learning rate set at 0.001, which gradually decreases as the training process progresses, and the learning rate is adjusted to one tenth of its previous level every 50 rounds. The advantage of this strategy

DOI: 10.2174/0115734056412035251027064309, 2026, *22*, e15734056412035

is that a higher learning rate in the early stages of training can accelerate the convergence speed of the model, while reducing the learning rate in the later stages of training can help the model stabilize near the optimal solution and avoid excessive oscillation.

REFERENCES

[1] Pasupa K, Vatathanavaro S, Tungjitnob S. Convolutional neural networks based focal loss for class imbalance problem: a case study of canine red blood cells morphology classification. Journal of Ambient Intelligence and Humanized Computing 2020; 1-17.

© 2026 The Author(s). Published by Bentham Science Publisher.

This is an open access article distributed under the terms of the Creative Commons Attribution 4.0 International Public License (CC-BY 4.0), a copy of which is available at: https://creativecommons.org/licenses/by/4.0/legalcode. This license permits unrestricted use, distribution, and reproduction in any medium, provided the original author and source are credited.

HOW TO CITE:
Li Y, Liu X, Zhang C, Qin X. A Deep Learning Radiomics Model Based on Superb Microvascular Imaging for Non-Invasive Prediction of the Degree of Arteriolosclerosis in Patients With Chronic Kidney Disease. Curr Med Imaging, 2026; 22: e15734056412035.
http://dx.doi.org/10.2174/0115734056412035251027064309
